# PHOX2B is a suppressor of neuroblastoma metastasis

**DOI:** 10.18632/oncotarget.7056

**Published:** 2016-01-28

**Authors:** Osnat Naftali, Shelly Maman, Tsipi Meshel, Orit Sagi-Assif, Ravit Ginat, Isaac P. Witz

**Affiliations:** ^1^ Department of Cell Research and Immunology, The George S. Wise Faculty of Life Sciences, Tel Aviv University, Tel Aviv, Israel 69978

**Keywords:** PHOX2B, minimal residual disease, metastasis, neuroblastoma, methylation

## Abstract

Paired like homeobox 2B (PHOX2B) is a minimal residual disease (MRD) marker of neuroblastoma. The presence of MRD, also referred to as micro-metastases, is a powerful marker of poor prognosis in neuroblastoma.

Lung metastasis is considered a terminal event in neuroblastoma. Lung micro-metastatic neuroblastoma (MicroNB) cells show high expression levels of PHOX2B and possess a less malignant and metastatic phenotype than lung macro metastatic neuroblastoma (MacroNB) cells, which hardly express PHOX2B.

*In vitro* assays showed that PHOX2B knockdown in MicroNB cells did not affect cell viability; however it decreased the migratory capacity of the MicroNB-shPHOX2B cells. An orthotopic inoculation of MicroNB-shPHOX2B cells into the adrenal gland of nude mice resulted in significantly larger primary tumors and a heavier micro-metastatic load in the lungs and bone-marrow, than when control cells were inoculated.

PHOX2B expression was found to be regulated by methylation. The PHOX2B promoter in MacroNB cells is significantly more methylated than in MicroNB cells. Demethylation assays using 5-azacytidine demonstrated that methylation can indeed inhibit PHOX2B transcription in MacroNB cells.

These pre-clinical data strongly suggest that PHOX2B functions as a suppressor of neuroblastoma progression.

## INTRODUCTION

Neuroblastoma is the most common extracranial solid tumor in children, and accounts for approximately 15% of all childhood cancer deaths [1, 2]. Most children diagnosed with neuroblastoma over the age of one year present metastatic disease, when the presence of lung metastases is considered a rare and terminal event [3, 4]. Most patients with metastatic disease who achieve near complete remission typically relapse because of the presence of neuroblastoma micro-metastasis, also known as minimal residual disease (MRD) [5–8]. Cure after clinical relapse is rare [9].

The current consensus is that micro-metastases remain in a dormant state, until “awakened” to progress towards overt metastases [10]. Understanding the mechanism regulating the progression of micro-metastases to overt metastasis is crucial for the development of efficient modalities to monitor and treat neuroblastoma metastasis.

The ideal MRD marker is one that is tumor specific and has no expression in the normal compartments. Stutterheim *et al.* (2008) found PHOX2B to be superior to TH and GD2 synthase, the commonly used MRD markers, in specificity and sensitivity of neuroblastoma MRD detection [11].

PHOX2B is a homeodomain transcription factor that promotes differentiation in neural crest cells [12]. PHOX2B was the first gene for which germline mutations - such as heterozygous missense and nonsense mutations - were found in patients with neuroblastoma [13, 14]. Subtyping neuroblastoma tumors indicated that low expression of PHOX2B is associated with higher tumor stage, poor outcome and poor survival [15].

We previously described the development of a mouse model for human neuroblastoma metastasis. An orthotopic inoculation of the human neuroblastoma cell line MHH-NB-11 [16] to the adrenal gland of athymic nude mice yielded local adrenal tumors, as well as lung metastasis. After several cycles of *in vivo* passages of cells cultured from these local tumors and lung metastases, local and lung metastatic variants were generated [17]. Nude mice inoculated orthotopically with neuroblastoma lung metastatic variants consistently generated overt lung macro-metastases, whereas mice inoculated orthotopically with local neuroblastoma variants generated lung micro-metastases but no macro-metastases[18]. Both the lung macro-metastatic and micro-metastatic cells were cultured yielding macro-metastatic (MacroNB) and micro-metastatic neuroblastoma (MicroNB) cell variants. These variants share the same genetic background.

The MicroNB cells were found to express significantly higher levels of the MRD marker PHOX2B, compared with the MacroNB cells which express no or very low levels of PHOX2B. Further characterization of these variants revealed that the MacroNB cells express a more malignant phenotype than the MicroNB cells [18].

In this study we asked if PHOX2B is involved in shaping the malignant and metastatic phenotype of neuroblastoma cells. We also investigated the mechanism regulating PHOX2B expression in MicroNB and MacroNB cells.

## RESULTS

### Downregulation of PHOX2B expression in MicroNB cells

**Figure 1:**
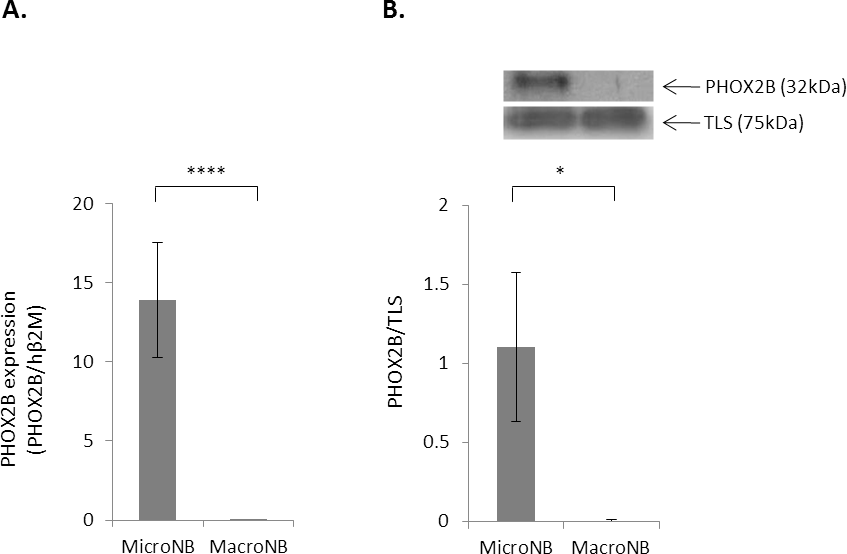
PHOX2B expression is higher in MicroNB than in MacroNB cells PHOX2B mRNA and protein levels were examined in the MicroNB and MacroNB cells. **A.** PHOX2B mRNA level in the MicroNB and MacroNB cells was examined by qRT-PCR and normalized to human β2M expression **B.** Nuclear cell lysates of MacroNB and MicroNB cells were subjected to western blot analysis. Specific antibodies were used for protein identification: anti-PHOX2B and anti-TLS (used as loading control). PHOX2B protein level was calculated in reference to TLS, as measured by densitometry. The blot presents a representative experiment of three independent ones. Data represent the mean ±SD of three independent experiments. Significance was evaluated using Student's *t*-test. *-*p*<0.05, ****-*p*<0.001.

In a previous study we found that MicroNB cells, but not MacroNB cells, express high mRNA levels of the MRD marker PHOX2B [18]. In this work, we confirmed this finding at the mRNA level by qRT-PCR (Figure 1A) and at the protein level by western blot (Figure 1B). The qRT-PCR results showed that PHOX2B expression in the MicroNB cells was more than 4 orders of magnitude greater (p<0.001) than in the MacroNB cells. Western blot analysis did not reveal any PHOX2B expression in the MacroNB cells (p<0.05).

**Figure 2:**
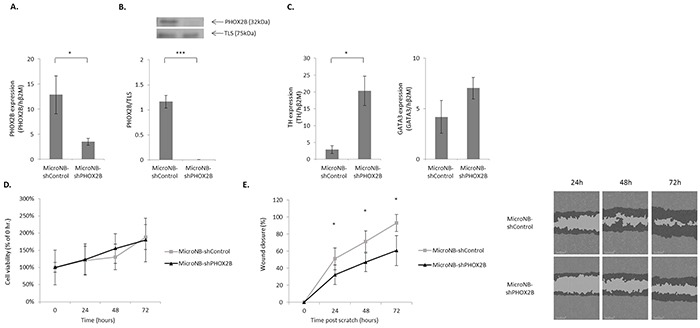
Downregulation of PHOX2B in MicroNB cells affects their malignant phenotype Downregulation of PHOX2B in the MicroNB variant influences neuroblastoma-associated-genes expression and cell-migration, but not cell-viability. **A.** PHOX2B mRNA level in the MicroNB-shControl and MicroNB-shPHOX2B cells was examined by qRT-PCR and normalized to human β2M. **B.** Nuclear cell lysates of MicroNB-shControl and MicroNB-shPHOX2B cells were subjected to western blot analysis. Specific antibodies were used for protein identification: anti-PHOX2B and anti-TLS (used as loading control). PHOX2B protein level was calculated in reference to TLS, as measured by densitometry. The blot presents a representative experiment of three independent ones. **C.** RNA extracted from MicroNB-shControl and MicroNB-shPHOX2B cells was used for qRT-PCR to measure changes in TH and GATA3 mRNA expression levels. All gene expression levels were normalized to human β2M. **D.** MicroNB-shControl and MicroNB-shPHOX2B cells were examined for their viability by an XTT-based assay at 0, 24, 48 and 72hr following seeding. Cell viability at each time point is presented relative to cell viability at 0hr. **E.** MicroNB-shControl and MicroNB-shPHOX2B cells were examined for their migratory capacity in a wound healing assay. Shown are representative photomicrographs of three independent experiments, taken at 24, 48 and 72hr post scratch. Image analysis was performed by IncuCyte software. Highlighted in black are cells that had migrated, and in light grey is the wound area that has not closed yet. Percentage of wound closure at every time point was calculated relatively to the wound width at 0hr. Data represent the mean ±SD of three independent experiments. Significance was evaluated using Student's *t*-test. *-*p*<0.05, ***-*p*<0.005.

To determine whether the differential expression of PHOX2B accounts for the differential malignant phenotype of MicroNB and MacroNB cells [18], we generated MicroNB cells in which PHOX2B expression was downregulated by PHOX2B specific shRNA (MicroNB-shPHOX2B). Control cells were infected with a non-silencing shRNA (MicroNB-shControl). qRT-PCR assays showed that following infection with PHOX2B-specific shRNA, PHOX2B mRNA expression decreased almost four fold (p<0.05) in the MicroNB-shPHOX2B cells (Figure 2A). Western blot analysis showed no expression of PHOX2B protein in the MicroNB-shPHOX2B cells (p<0.005) (Figure 2B).

### PHOX2B downregulation alters the expression of neuroblastoma-associated genes

The influence of PHOX2B downregulation on the malignant phenotype of the cells was first evaluated by measuring expression levels of Tyrosine hydroxylase (TH) and GATA binding protein 3 (GATA3) which are genetically downstream to PHOX2B and have been linked with an unfavorable outcome and oncogenicity, respectively [19–23]. qRT-PCR measurements indicated that downregulation of PHOX2B significantly (p<0.05) increased TH expression. No significant change in GATA3 expression was observed (Figure 2C). The increased TH expression, a marker for bad prognosis in neuroblastoma [22, 24], suggested an involvement of PHOX2B in shaping the malignant phenotype of neuroblastoma cells.

### PHOX2B downregulation affects the malignant phenotype of the cells

If expression levels of PHOX2B are indeed related to the malignant phenotype of neuroblastoma cells, it is to be expected that knockdown of PHOX2B would promote the malignant properties of these cells. This hypothesis was examined in the following set of experiments.

XTT-based viability assays were performed in order to determine the influence of PHOX2B down-regulation on MicroNB cell viability. MicroNB-shControl and MicroNB-shPHOX2B cell viability was examined following 0, 24, 48 and 72hr growth under normal conditions. Our results showed no significant difference in cell viability between the MicroNB-shControl and MicroNB-shPHOX2B cells (Figure 2D).

We previously found that MicroNB cells have a significantly lower migratory capacity than MacroNB cells [18]. Here, we examined whether the low migratory capacity of MicroNB cells is associated with the high PHOX2B expression levels of these cells. Using the wound healing assay it was found that PHOX2B knockdown significantly (p<0.05) impaired the ability of the cells to migrate and close the wound: only 32%, 47% and 61% wound closure at 24, 48 and 72hr respectively was measured for the MicroNB-shPHOX2B cells, compared with 51%, 71% and 93% closure measured for the MicroNB-shControl cells (Figure 2E). This result cannot be attributed to different proliferation rates of MicroNB-shPHOX2B and MicroNB-shControl cells since no such difference was observed (Figure 2D). It shows, however, that PHOX2B restrains the migratory capacity of neuroblastoma cells.

### PHOX2B knockdown increases tumorigenicity and lung and bone-marrow metastasis

**Figure 3:**
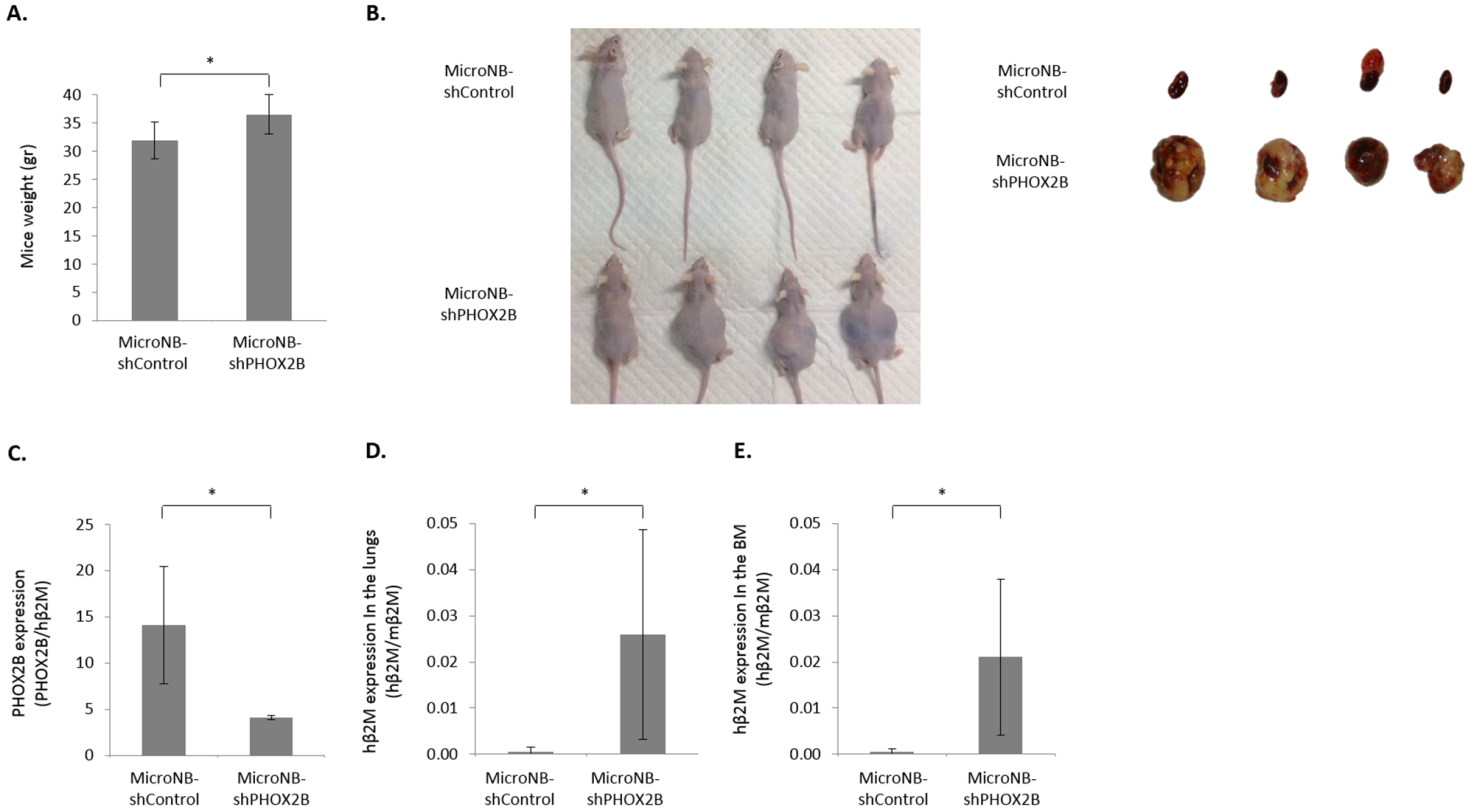
Downregulation of PHOX2B increases tumorigenicity and metastasis Nude mice to which MicroNB-shControl and MicroNB-shPHOX2B cells were orthotopically inoculated were sacrificed when moribund, and primary tumors, bone-marrow and lungs were extracted for RNA quantitation. **A.** Mice were weighed weekly. Presented is the average weight of mice when sacrificed. **B.** A representative photo presenting mice and primary tumors, four of each group. **C.** RNA samples extracted from primary tumors were used to measure PHOX2B mRNA expression: PHOX2B mRNA levels were normalized to the human β2M expression levels. **D, E.** RNA samples extracted from lungs (D) and bone-marrow (E) were used to measure micro metastatic burden, by measuring human β2M mRNA expression level. Human β2M mRNA levels were normalized to the mouse β2M expression levels. Data represent the mean (*n*=10, 5 mice in each group) ± SD. Significance was evaluated using Student's *t*-test. *-*p*<0.05.

To establish whether PHOX2B expression influences the tumorigenic and metastatic phenotype of MicroNB cells, we orthotopically inoculated the MicroNB-shControl and MicroNB-shPHOX2B cells to the adrenal gland of nude mice. Mice were weighed weekly. At the time of sacrifice, mice inoculated with MicroNB-shPHOX2B cells weighed significantly more than mice inoculated with MicroNB-shControl cells (p<0.05) (Figure 3A); The increased weight of the mice inoculated with MicroNB-shPHOX2B cells was probably due to their heavier tumor and metastatic load (Figure 3B).

Local tumors, lungs and bone-marrow were evaluated for tumor load and PHOX2B expression. At the time of sacrifice, no overt lung macro-metastases were seen. A significant difference was observed in the local tumor size: Mice inoculated with MicroNB-shPHOX2B cells developed larger local tumors than MicroNB-shControl-inoculated mice (Figure 3B). qRT-PCR performed with RNA extracted from the local tumors, established that the tumors maintained the original PHOX2B expression of the injected cells: Cells harvested from local tumors of MicroNB-shControl- inoculated mice expressed higher levels of PHOX2B than cells harvested from local tumors of MicroNB-shPHOX2B- inoculated mice (p<0.05) (Figure 3C).

Since no overt metastases were apparent at the time of sacrifice, a qRT-PCR was performed to evaluate the presence of micro-metastases in the lungs and bone-marrow of the inoculated mice. The results indicated that the knockdown of PHOX2B in MicroNB cells increased significantly their micro-metastatic load. A 50 and 40 fold higher load (p<0.05) was detected respectively in the lungs and bone-marrow of mice bearing MicroNB-shPHOX2B tumors than in the lungs and bone-marrow of control mice (Figure 3D, 3E). This result supports our previous finding that MacroNB cells expressing no or very low levels of PHOX2B, produce a higher metastatic burden than high PHOX2B expressing MicroNB cells [18]. More importantly, this indicates that PHOX2B is a regulator of neuroblastoma progression and metastasis.

### The mechanism regulating the differential expression of PHOX2B in MicroNB and MacroNB cells

To investigate the mechanism responsible for the differential expression of PHOX2B in the MicroNB and MacroNB variants, we sequenced the PHOX2B gene and examined the role of methylation in PHOX2B gene expression, as detailed in the following set of experiments.

### PHOX2B sequence is identical in the MicroNB and MacroNB cells

**Figure 4:**
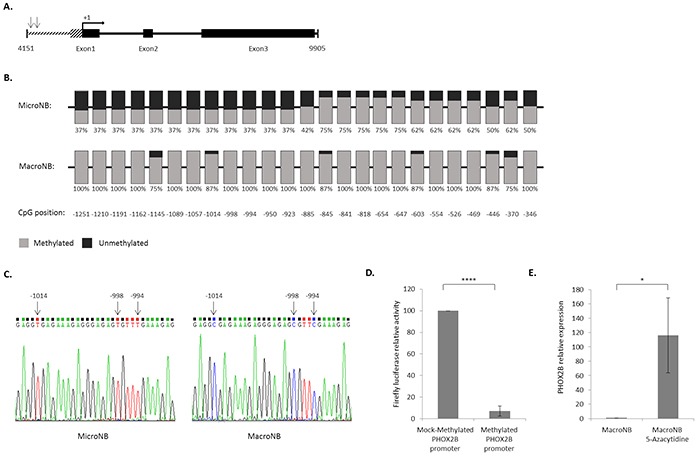
High methylation level of the PHOX2B promoter reduces its expression The genomic sequence and promoter methylation pattern of PHOX2B gene, as well as the effect of promoter methylation on gene transcription, were examined in MicroNB and MacroNB cells. **A.** Genomic DNA extracted from MicroNB and MacroNB cells was used for sequencing of a 5754bp long fragment containing the region spanning from the core PHOX2B promoter up to the end of exon 3 (positions 4151- 9905; NG_008243.1). Striped area marks the core PHOX2B promoter, arrows mark point mutations: 4181A → C and 4194C → T, the +1 arrow symbolizes the transcription start site [27]. **B.** Genomic DNA extracted from MicroNB and MacroNB cells was used in bisulfite sequencing analysis (25 CpG dinucleotides along a 1061bp sequence of the PHOX2B promoter). CpG positions are marked relatively to the +1 transcription start site [27]. Indicated below each CpG position is its methylation status. **C.** Representative sequencing results of the PHOX2B promoter in MicroNB and MacroNB cells following bisulfite conversion. **D.** PHOX2B Promoter activity was measured by a luciferase reporter assay. Firefly luciferase reporter gene activity was determined by transient transfection of SK-NMC cells with pGL2-PHOX2B-MOCK (Mock-Methylated PHOX2B promoter) or pGL2-PHOX2B-MET (Methylated PHOX2B promoter) vectors. The graph shows Firefly luciferase activity after normalization to Renilla luciferase activity and relative to the activity measured in Mock-Methylated PHOX2B promoter. **E.** Following a 96hr treatment with the demethylation reagent 5-Azacytidine, PHOX2B mRNA expression in MacroNB cells and in MacroNB 5-Azacytidine treated cells was examined by qRT-PCR and normalized to RPS13 expression. The graph shows PHOX2B normalized expression relative to the expression measured in MacroNB cells. Results represent the mean ±SD of three independent experiments. Significance was evaluated using Student's *t*-test. *-*p*<0.05, ****-*p*<0.001.

To exclude the possibility that heterozygous mutations of the PHOX2B gene [25] are the cause of the PHOX2B differential expression in the MicroNB and MacroNB cells, we sequenced the PHOX2B gene in these two cell variants. A 5754bp long fragment containing the region spanning from the core PHOX2B promoter up to the end of exon 3 was isolated from the MicroNB and MacroNB cells by PCR and sequenced. It was found that both cells share an identical sequence. Also, two point mutations (4181A→C and 4194C→T; numbered according to RefSeq accession no.: NG_008243.1) were found in the PHOX2B promoter region of both the MicroNB and the MacroNB cells. However, these mutations were neither located in a CpG dinucleotide or in a known transcription factor binding site [26, 27] (Figure 4A). This determined that the differential expression of PHOX2B in MicroNB and MacroNB cells is not derived from changes in its genomic sequence.

### The PHOX2B promoter is highly methylated in MacroNB cells

PHOX2B promoter methylation leads to PHOX2B inactivation in neuroblastoma tumors and cell lines [28]. In order to establish whether promoter methylation is responsible for the differential expression of PHOX2B in MicroNB and MacroNB cells, we mapped the methylation pattern of the PHOX2B promoter in these cells. We first performed a bisulfite modification of the genome of the cells followed by PCR isolation of four amplicons containing fragments of the PHOX2B promoter (along 1061bp): amplicon I and II generated from the MicroNB converted genome, and amplicon I and II generated from the MacroNB converted genome. Eight clones randomly selected from each amplicon were used to examine the methylation status (the percentage of methylation in each CpG dinucleotide) of the PHOX2B promoter in each variant. Sequencing analysis of these clones showed a 75-100% methylation along 25 CpG dinucleotides in the PHOX2B promoter region of MacroNB cells compared with only 37-75% methylation of these CpG dinucleotides in MicroNB cells (Figure 4B). A representative sequencing result shows how three Cytosine nucleotides (CpG positions: −1014, −998 and −994) are unmethylated in MicroNB cells, thus appearing as Thymine in the DNA, while the same nucleotides are methylated in the MacroNB cells, thus appearing as Cytosine (Figure 4C). These results strongly suggest methylation as the cause for PHOX2B lack of expression in the MacroNB cells.

### *In vitro* methylation of the PHOX2B promoter diminishes transcription

To further establish that methylation of the PHOX2B promoter is able to prevent gene transcription, we performed a luciferase reporter assay. The core PHOX2B promoter (a 1.3kb sequence located upstream to the PHOX2B transcription start site) which was found to be sufficient for PHOX2B transcription [27] was cloned upstream to a Firefly luciferase gene in a pGL2 vector, to create a pGL2-PHOX2B vector. We then either methylated or mock-methylated the vector, and examined the Firefly luciferase transcription levels in transfected SK-NMC neuroblastoma cells. Renilla luciferase vector was used as an internal control. In cells transfected with the vector carrying the methylated promoter (Methylated PHOX2B promoter) 93% less transcription (P<0.001) of the Firefly luciferase was detected, compared with transcription levels measured in cells transfected with the vector carrying the mock-methylated promoter (Mock-Methylated PHOX2B promoter) (Figure 4D). This result provides proof that methylation of the PHOX2B promoter is indeed capable of inhibiting gene transcription.

### Treatment with the demethylating agent 5-Azacytidine induces PHOX2B expression

The demethylation reagent 5-Azacytidine incorporates into the DNA and inhibits DNA methylation [29]. We treated MacroNB cells, which are highly methylated in the PHOX2B promoter area (Figure 4B), with 5-Azacytidine for 96hr and measured PHOX2B mRNA levels by qRT-PCR. We found that following treatment, the relative PHOX2B mRNA expression was 100 fold elevated (Figure 4E), a direct evidence that PHOX2B expression in MacroNB cells is suppressed by methylation.

## DISCUSSION

PHOX2B, a transcription factor and a specific and sensitive bio-marker for MRD in neuroblastoma patients [11, 12], is highly expressed in micro-metastatic neuroblastoma cells (MicroNB), but not, or significantly less in macro-metastatic cells (MacroNB). These cells also differ in their malignant and metastatic phenotype; the latter cells form lung macro-metastases, while the MicroNB cells generate only lung micro-metastases [18]. This study is based on the well-established fact that overt metastasis in a specific organ develops from precursor micro-metastatic cells residing in it [10, 30–34]. The main goal of this research was, therefore, to find out whether PHOX2B is functionally involved in shaping the micro-metastatic phenotype of neuroblastoma cells.

In the present study we established that PHOX2B functions as a suppressor of neuroblastoma metastasis: The knockdown of PHOX2B in human neuroblastoma micro-metastatic cells increased the tumorigenic and metastatic potential of the cells in orthotopically inoculated nude mice. As reported above, the PHOX2B-mediated downregulation induced an upregulation of TH, a marker for bad prognosis in neuroblastoma [22]. It is still to be determined whether the upregulation of TH is involved in promoting the malignant behavior of neuroblastoma cells.

There was a discrepancy between the *in vivo* results indicating that the knockdown of PHOX2B promoted local tumor formation and increased metastatic load, and the *in vitro* results demonstrating that PHOX2B knockdown did not affect proliferation and decreased the migratory capacity of the cells. Discrepancies between *in vivo* and *in vitro* findings are rather common [35–38] and are to be expected. Tumors do not proliferate or progress without cross-talk with various types of stromal cells and their products [39, 40]. Tumorigenesis and metastasis of neuroblastoma cells *in vivo* are regulated by a balanced function of positive and negative signals [10] including those regulated by PHOX2B. The *in vitro* milieu, in which proliferation and migration of shPHOX2B neuroblastoma cells were measured, did not provide these additional signals.

Tumor progression is largely regulated by the tumor microenvironment [30, 41–43] and different microenvironments differently control gene expression of tumor cells [44–46]. Since the MicroNB and MacroNB variants share a common genetic background but initially originated from different microenvironments (the MicroNB cells originated from adrenal tumors and the MacroNB cells originated from lung metastasis) [17, 18], it is not unlikely that the differential expression of PHOX2B in these cells is due to genetic or epigenetic events that took place in the different microenvironments from which these variant progenitors were generated.

DNA hyper-methylation is an epigenetic event occurring in many cancer types [47–50] and one that plays a role in PHOX2B silencing in neuroblastoma cell-lines and tumors [28]. We found that the PHOX2B promoter, that has an identical sequence in MicroNB and MacroNB cells, is more highly methylated in the MacroNB cells than in the MicroNB cells. It was also established that methylation of the PHOX2B promoter is able to reduce transcription by 93%. Treatment with the demethylating agent 5-azacytidine induced PHOX2B expression in the MacroNB cells, verifying that the low PHOX2B expression in the MacroNB cells is indeed due to high methylation of its gene in these cells.

Taken together this pre-clinical study demonstrates that PHOX2B is a metastasis suppressor, able to inhibit the tumorigenic and metastatic abilities of neuroblastoma cells and that this inhibitory function is likely mediated by the tumor microenvironment via an epigenetic mechanism.

## MATERIALS AND METHODS

### Cell culture

The human neuroblastoma lung micro-metastatic (MicroNB) and macro-metastatic (MacroNB) variants were generated using a mouse model for human neuroblastoma metastasis [17, 18] from the parental cell line MHH-NB-11 [16] and were maintained in culture as previously described [17]. SK-NMC cells were maintained in culture as previously described [51]. MicroNB-shControl and MicroNB-shPHOX2B cells were maintained as monolayer cultures in growth medium: RPMI 1640 medium supplemented with 10% fetal calf serum (FCS), 100 U/ml streptomycin, 12.5 U/ml nystatin, 100 U/ml penicillin, 2 mM L-glutamine (all materials were purchased from Biological Industries, Beit Ha'emek, Israel), and 2 μg/ml puromycin (InvivoGen, San Diego, CA, USA). The cultures were incubated at 37°C in a mixture of 6.5% carbon dioxide. Cell authentication was performed by short tandem repeat analysis of DNA using the Type-it Microsatellite PCR Kit (Qiagen, Valencia, CA, USA), for the genes published by the ATCC (Manassas, VA, USA). All cultures were periodically examined for mycoplasma contamination.

### Animals

Male athymic nude mice (BALB/c background) were purchased from Harlan Laboratories (Jerusalem, Israel). The mice were housed and maintained as previously described [52]. All experiments involving animals were approved by the TAU Institutional Animal Care and Use Committee. Mice of 7–10 weeks old were used for experiments in accordance with institutional ethical guidelines.

### qRT-PCR

Total RNA was extracted from cell cultures using the EZ-RNA Total RNA Isolation Kit (Biological Industries) and used in quantitative real-time PCR (qRT-PCR) as previously described [18]. From harvested organs, total RNA was extracted using the Bullet blender bead lysis kit (Next advance Inc., Averill Park, NY, USA) and the EZ-RNA Total RNA Isolation Kit (Biological Industries). Detection of human neuroblastoma cells (micro-metastases) in mouse tissue was performed as previously described [18], using specific primers for the human/mouse β2 microglobulin. Primers used for mRNA amplification were: human β2 microglobulin (NM_004048) For-5′-ATGTAAGCAGCATCATGGAG-3′, Rev-5′-AAGCAAGCAGAATTTGGAAT-3′; mouse β2 microglobulin (NM_009735.3) For-5′-CTGGTCTTTCTGGTGCTTGT-3′, Rev- 5′-GGCGTGAGTATACTTGAATTTGAG-3′; PHOX2B (NM_003924.3) For-5′-TACGCCGCAGTTCCTTACAA-3′, Rev-5′-GAAGACCCTTTCCAGCTCTTT-3′; TH (NM_000360.3) For-5′-TGTACTGGTTCACGGTGGAGTT-3′, Rev-5′-AATCTCAGGCTCCTCAGACA-3′; GATA3 (NM_001002295.1) For-5′-ACACTCTGGAGGAGGAATGC-3′, Rev-5′-CTGGATGCCTTCCTTCTTCATA-3′; RPS13 (NM_001017.2) For-5′-CGAAAGCATCTTGAGAGGAACA-3′, Rev-5′-TCGAGCCAAACGGTGAATC-3′.

### Western blotting

Neuroblastoma cells were seeded in 60mm plates (1×10^6^ cells/plate) in normal growth conditions. 48hr following seeding, cells were washed twice with PBSx1 (Biological Industries) and stored in −70°c until use. Cell pellets were lysed with buffer lysis (5mM Hepes pH 7.9, 1mM Na3VO4, 1mM NaPPi, 1mM NaF, 1mM PMSF, 2μg/ml Leupeptin, 2μg/ml Aprotinin, 1% NP-40) then centrifuged for 20min in 16,000g 4°c. For nuclear fractionation, the upper layer was removed and the remaining pellet was washed (20mM Hepes pH 7.9, 5mM KCl, 150mM NaCl), then centrifuged for 10min in 16,000g, 4°c. The upper layer was removed and the remaining pellet was lysed with buffer lysis (20mM Hepes pH 7.9, 420mM NaCl, 1mM EDTA, 1mM EGTA, 1mM dithiothreitol, 1mM PMSF, 1mM Na3VO4, 4mM β glycerol phosphate, 2μg/ml Leupeptin, 2μg/ml Aprotinin), then ice incubated for 30min and centrifuged for 10min in 16,000g, 4°c. The upper layer was used as the nuclear lysate, and protein concentration in the samples was determined by BCA Protein Assay reagent (Thermo Fisher Scientific, Waltham, MA, USA). Samples were subjected to western blot analysis as previously described [18]. Goat polyclonal anti-PHOX2B (1:200, Santa Cruz Biotechnology Inc., Santa Cruz, CA, USA) and Rabbit polyclonal anti-TLS/FUS (1:30,000, Abcam, Cambridge, UK) were used in PHOX2B and TLS detection, respectively. HRP-conjugated donkey anti-goat IgG and HRP-conjugated goat anti-rabbit IgG (Jackson immunosearch laboratories, West Grove, PA, USA) were used as secondary antibodies, respectively, according to the manufacturer's instructions.

### Downregulation of PHOX2B expression

The downregulation of PHOX2B was constructed using pGIPZ vectors (Thermo Fisher Scientific). Seven different pGIPZ vectors containing seven different shRNA sequences targeting PHOX2B mRNA (NM_003924.3) were examined for their downregulation efficiency using qRT-PCR and western blotting. Two of the seven vectors which showed the strongest effect (RHS4430-98843809, RHS4430-101168343) were then used for PHOX2B downregulation in the MicroNB variant (MicroNB-shPHOX2B). A sh-non-silencing pGIPZ vector (RHS4531) was used as a negative control (MicroNB-shControl).

To produce the infectious viruses, the 293T packaging cell line was co-transfected using a calcium phosphate method with the lentiviral backbone plasmids shPHOX2B-pGIPZ or the sh-non-silencing-pGIPZ, packaging plasmid pCMVΔR8.2 and envelope plasmid pVSV-G. After 48hr, the virus particles in the medium were collected and filtrated (0.45 mm, Whatman GmbH, Freiburg, Germany). A total of 1×10^6^ MicroNB cells, seeded 24hr before infection, were infected overnight in the presence of 8 μg/ml polybrene and the virus-containing medium, which was afterwards replaced with fresh growth medium. After 72hr 2 μg/ml puromycin (InvivoGen) was added for additional 7 days to select stably infected cell population. After selection, puromycin was continuously added to the culture.

### *In vitro* viability assay

MicroNB-shControl and MicroNB-shPHOX2B cells were seeded (1×10^4^ per well) in a 96-well, flat-bottomed, tissue culture plate. Viability under normal growth conditions [17] was monitored in triplicates using an XTT-based viability assay after 0, 24, 48, and 72 hours, according to the manufacturer's instructions (Biological Industries). Cell viability was determined as previously described [18].

### Wound healing assay

MicroNB-shControl and MicroNB-shPHOX2B cells were seeded onto a 96-well ImageLock tissue culture plate (Essen BioScience, Ann Arbor, MI, USA) which was pre-coated with 0.015% Poly-L-lysin (Sigma-Aldrich Corp., St Louis, MO, USA). Cells were allowed to attach over a 24hr period, then wounds were made with the 96-well WoundMaker (Essen BioScience). The culture plate was washed once with PBSx1 to remove detached cells, and a fresh growth medium was added. Images of the wounds were automatically acquired within the CO_2_ incubator by IncuCyte zoom (Essen BioScience). Typical photomicrographs were taken at 2hr intervals for a 72hr period. The data were analyzed with respect to wound confluence and calculated using the IncuCyte software (Essen BioScience).

### Orthotopic inoculation of tumor cells

An orthotopic inoculation to the adrenal gland of athymic nude mice was performed with 1×10^6^ MicroNB-shControl or MicroNB-shPHOX2B cells suspended in 50μl growth medium (RPMI-1640 containing 5% fetal calf serum (FCS); Biological Industries). The intra-adrenal inoculation required surgical exposure of the left adrenal gland under anesthesia and was performed as previously described [17]. We weekly monitored local tumor development and mice weights. Mice were sacrificed when moribund (about 90 days after inoculation) and local adrenal tumor, lungs and sternum (bone marrow) were harvested. The organs were immediately flash-frozen on dry ice, then stored at −70°c until used for RNA extraction.

### Sequencing of the PHOX2B gene

Genomic DNA was extracted from the MicroNB and MacroNB cells using QIAamp DNA Mini Kit (Qiagen). We custom designed primers covering a 5754bp sequence containing the region spanning from the core PHOX2B promoter up to the end of exon 3 (positions 4151- 9905; NG_008243.1). The primers for amplicon I are: For- 5′-TACAGTCCGCAAACCTAAAAGG-3′, Rev- 5′-CTAAGCTTTACGTCTCATCGCA-3′, for amplicon II: For- 5′-TACGCCGCAGGTAAGGACC-3′, Rev- 5′-GTCAGTGCTCTTGGCCTCTT-3′, for amplicon III: For- 5′-GCAAAGAGGCCAAGAGCACTGA-3′, Rev- 5′-TTATCAATGCCCTGGTGTGCTTCT-3′. Amplicons were PCR amplified using the KAPA HiFi PCR Kit (Kapa Biosystems, Boston, MA, USA) and sequenced in the ABI 3500xl Genetic analyzer (Life technologies, Waltham, MA, USA). PCR conditions were: 39 cycles of: 98°c for 20sec, 68°c /69°c/68°c for 15sec, 72°c for 1.50min/1.20min/3.40min, for amplicons I, II and III respectively.

### Bisulfite conversion and methylation pattern analysis of the PHOX2B promoter

Genomic DNA was extracted from the MicroNB and MacroNB cells using QIAamp DNA Mini Kit (Qiagen). 500ng of sample DNA was treated with sodium bisulfite using the EZ DNA Methylation-lightning Kit (Zymo Research, Irvine, CA, USA). Treatment was according to the manufacturer's instructions, only that incubation with L-Desulphonation Buffer was performed in 30°c. We custom designed primers for two amplicons spanning the core PHOX2B promoter, covering a 1061bp long sequence in total, starting 1322bp upstream to the first transcription start site [27]. The primers for amplicon I are: For- 5′-AGAGGAAGGATTTAATGAAATAGT-3′ and Rev- 5′-CCRTTATATTCTTTACTCTAATTCTATCTATTTAC-3′. For amplicon II: For- 5′-TTGYGTTAATGTAAATAGATAGAATTGAGAG-3′ and Rev- 5′-CACCRAACCCCTAATCCTCCCTTCTAACCAAC-3′. These primers were used in PCR with GoTaq DNA polymerase (Promega, Madison, WI, USA), with 3mM Mg^2+^ and an annealing temp of 56°c. Thus we generated four PCR products: amplicon I and II generated from the MicroNB converted genome, and amplicon I and II generated from the MacroNB converted genome. Each PCR product was cloned into the pCRII-TOPO vector (Invitrogene, Waltham, MA, USA), then transformed to XL1 blue bacteria and grown on an Amp+Tet agarose plates. Eight clones were randomly selected from each of the PCR products and sequenced in the ABI 3500xl Genetic analyzer (Life technologies) to detect methylation level of each CpG dinucleotides. Sequencing results data were analyzed by the Chromas LITE software (Technelysium, South Brisbane, Australia).

### Luciferase assay

A 1300bp amplicon containing the core PHOX2B promoter (positions 3948- 5245; NG_008243.1) was isolated from the MacroNB variant by DNA extraction using the QIAamp DNA Mini Kit (Qiagen) followed by PCR using the KAPA HiFi PCR Kit (Kapa Biosystems). Forward and reverse primers contained either a SacI or Bgl2 recognition sites, respectively (For- 5′-ATATATGAGCTCTTCCCTTATTCTGCTAGGGCCTAA-3′, Rev- 5′-AATAATAGATCTCCTTACTACCAGCAGAGCCTAGTTT-3′). PCR conditions were: 2 cycles of: 98°c for 30sec, 60°c for 15sec, 72°c for 1.30min followed by 43 cycles of: 98°c for 30sec, 68°c for 15sec, 72°c for 1.5min. The amplicon was cloned into a pGL2-basic vector (promega) which contains luciferase as a reporter gene. The vector was then either methylated/mock-methylated by SssI methylase (New England Biolabs, Ipswich, MA, USA) thus generating a pGL2-PHOX2B-MET or pGL2-PHOX2B-MOCK vector, respectively. The procedure for mock-methylation was identical except no SssI methylase was included. SK-NMC cells were seeded in a 24 well plate, allowed to attach over a 24hr period, and transfected with either pGL2-PHOX2B-MET or a pGL2-PHOX2B-MOCK vectors, and with the Renilla luciferase pRL-TK vector (promega). Transfection was performed O.N. using Lipofectamin 2000 and OptiMem reduced serum medium (invitrogne). 24hr following transfection SK-NMC-pGL2-PHOX2B-MET and SK-NMC-pGL2-PHOX2B-MOCK cells were removed by Trypsin-B (Biological Industries), washed twice with PBSx1 (Biological Industries) and pellets were stored in −70°c. Pellets were subjected to luciferase assay using the Dual-Luciferase Reporter Assay System (promega) in the Veritas microplate luminometer (Turnerbiosystems, Sunnyvale, CA, USA). Results were then analyzed with the Veritas Microplate Luminometer software (Turnerbiosystems).

### 5-Azacytidine treatment

MacroNB cells were seeded in 60mm plates (7×10^5^ cells/plate) and allowed to attach over a 24hr period. Cells were then treated with 5μM (dissolved in 50% acetic acid) of the demethylation reagent 5-Azacytidine (Sigma-Aldrich Corp.) for 72hr, then growth medium was replaced with fresh medium and 5μM of 5-Azacytidine were added for another 24h period. After the 96hr treatment RNA was extracted from the cells and qRT-PCR was performed (see qRT-PCR in this section).

### Statistical analysis

Paired or unpaired Student's *t*-test was used to compare *in vitro* and *in vivo* results.
